# Isolation and Characterization of Multi-Protein Complexes Enriched in the K-Cl Co-transporter 2 From Brain Plasma Membranes

**DOI:** 10.3389/fnmol.2020.563091

**Published:** 2020-10-22

**Authors:** Joshua L. Smalley, Georgina Kontou, Catherine Choi, Qiu Ren, David Albrecht, Krithika Abiraman, Miguel A. Rodriguez Santos, Christopher E. Bope, Tarek Z. Deeb, Paul A. Davies, Nicholas J. Brandon, Stephen J. Moss

**Affiliations:** ^1^Department of Neuroscience, Tufts University School of Medicine, Boston, MA, United States; ^2^AstraZeneca Tufts Lab for Basic and Translational Neuroscience, Boston, MA, United States; ^3^Neuroscience, IMED Biotech Unit, AstraZeneca, Boston, MA, United States; ^4^Department of Neuroscience, Physiology, and Pharmacology, University College London, London, United Kingdom

**Keywords:** potassium chloride cotransporter 2, proteome, interactome, phosphorylation, protein purification

## Abstract

Kcc2 plays a critical role in determining the efficacy of synaptic inhibition, however, the cellular mechanisms neurons use to regulate its membrane trafficking, stability and activity are ill-defined. To address these issues, we used affinity purification to isolate stable multi-protein complexes of K-Cl Co-transporter 2 (Kcc2) from the plasma membrane of murine forebrain. We resolved these using blue-native polyacrylamide gel electrophoresis (BN-PAGE) coupled to LC-MS/MS and label-free quantification. Data are available via ProteomeXchange with identifier PXD021368. Purified Kcc2 migrated as distinct molecular species of 300, 600, and 800 kDa following BN-PAGE. In excess of 90% coverage of the soluble N- and C-termini of Kcc2 was obtained. In total we identified 246 proteins significantly associated with Kcc2. The 300 kDa species largely contained Kcc2, which is consistent with a dimeric quaternary structure for this transporter. The 600 and 800 kDa species represented stable multi-protein complexes of Kcc2. We identified a set of novel structural, ion transporting, immune related and signaling protein interactors, that are present at both excitatory and inhibitory synapses, consistent with the proposed localization of Kcc2. These included spectrins, C1qa/b/c and the IP3 receptor. We also identified interactors more directly associated with phosphorylation; Akap5, Akap13, and Lmtk3. Finally, we used LC-MS/MS on the same purified endogenous plasma membrane Kcc2 to detect phosphorylation sites. We detected 11 sites with high confidence, including known and novel sites. Collectively our experiments demonstrate that Kcc2 is associated with components of the neuronal cytoskeleton and signaling molecules that may act to regulate transporter membrane trafficking, stability, and activity.

## Introduction

The K^+^/Cl^–^ co-transporter Kcc2 (encoded by the gene *SLC12A5*) is the principal Cl^–^-extrusion mechanism employed by mature neurons in the CNS (Moore et al., 2017). Its activity is a pre-requisite for the efficacy of fast synaptic inhibition mediated by Glycine (GLYR) and type A γ-aminobutyric acid receptors (GABA_*A*_R), which are Cl^–^ permeable ligand-gated ion channels. At prenatal and early postnatal stages in rodents, neurons have elevated intracellular Cl^–^ levels resulting in depolarizing GABA_*A*_-mediated currents (Ben-Ari et al., 2012). The postnatal development of canonical hyperpolarizing GABA_*A*_R currents is a reflection of the progressive decrease of intraneuronal Cl^–^ levels that is caused by the upregulation of Kcc2 expression and subsequent activity (Payne, 1997; Rivera et al., 1999; Li et al., 2002; Uvarov et al., 2006). These changes in neuronal Cl^–^-extrusion reflect a sustained increase in the expression levels of Kcc2 after birth, the mRNA levels of which do not reach their maximal levels in humans until 20–25 years of age (Sedmak et al., 2016). In addition to this, the appropriate developmental appearance of hyperpolarizing GABA_*A*_R currents is also in part determined by the phosphorylation status of Kcc2, a process that facilitates its membrane trafficking and activity (Lee et al., 2007, 2011; Rinehart et al., 2009; Titz et al., 2015; Moore et al., 2019; Pisella et al., 2019).

In keeping with its essential role in determining the efficacy of synaptic inhibition, humans with mutations in Kcc2 develop severe epilepsy soon after birth (Stödberg et al., 2015; Saitsu et al., 2016; Saito et al., 2017). Deficits in Kcc2 activity are also believed to contribute to the development of temporal lobe epilepsy (Chen et al., 2017; Kelley et al., 2018), in addition to other traumas including ischemia and neuropathic pain (Jaenisch et al., 2010; Mapplebeck et al., 2019). Given the critical role that Kcc2 plays in determining the maturation of inhibitory neurotransmission, subtle changes in its function are also strongly implicated in autism spectrum disorders (ASD) (Tyzio et al., 2014), Down syndrome (Deidda et al., 2015), fragile X syndrome (He et al., 2014), and Rett syndrome (Duarte et al., 2013; Tang et al., 2016, 2019).

Here, we isolated and resolved stable protein complexes that contain Kcc2 from highly purified plasma membranes from mouse forebrain, and characterized their composition using a quantitative proteomic approach. In this way we were able to detect Kcc2 interactors in rank order of abundance. We subsequently identified several novel Kcc2-associated proteins. We used the same purification strategy and proteomics to measure Kcc2 phosphorylation. In this way we have developed new insights into the potential mechanisms of Kcc2 stabilization and regulation in the plasma membrane along with measuring the phosphorylation status of Kcc2 on the cell surface in parallel.

## Results

### Isolating Native Stable Protein Complexes Containing Kcc2 From Brain Plasma Membranes

**FIGURE 1:**
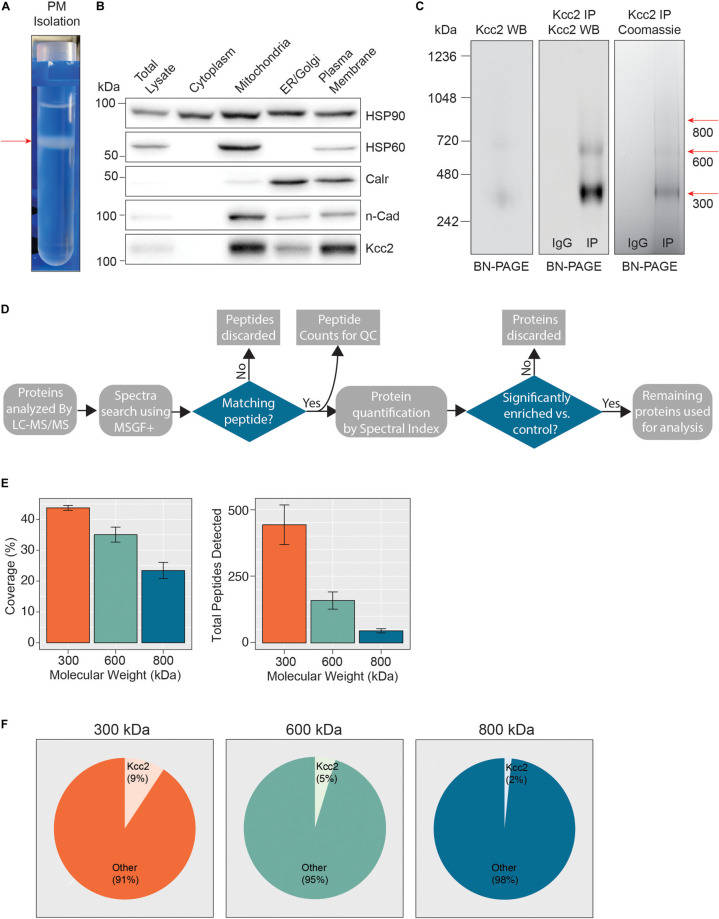
Highly enriched Kcc2-containing protein complexes were isolated from purified plasma membranes from mouse forebrain. **(A)** Differential centrifugation of homogenized forebrain from 8 to 12-week-old mice was used to fractionate cellular organelle and obtain an enriched plasma membrane fraction, visualized here at the final purification stage on a discontinuous sucrose gradient. **(B)** Western blots of the organelle fractions were used to measure the abundance of organelle markers; HSP90 (cytosol), HSP60 (mitochondria), calreticulin (ER/Golgi), n-Cadherin (n-Cadh) (plasma membrane). Kcc2 abundance was also measured to confirm Kcc2 enrichment in the plasma membrane fraction. **(C)** Blue-native PAGE (BN-PAGE) was carried out on plasma membrane lysates, and immunoprecipitated Kcc2 to resolve the native protein complexes that contain Kcc2. These were correlated with protein bands observed by Coomassie staining. Coomassie-stained bands were excised and used for proteomic analyses. **(D)** Flow diagram of the methodological approach for proteomic data processing. **(E)** Detected peptides were mapped to the Kcc2 reference sequence to determine Kcc2 sequence coverage obtained from LC-MS/MS of BN-PAGE protein bands produced following Kcc2 IP. Bar charts of the Kcc2 sequence coverage expressed as a percentage of the full sequence and by total Kcc2 peptides detected (*n* = 4). **(F)** Pie charts showing the average number of total Kcc2 peptides detected by LC-MS/MS relative to the average number of total peptides detected for all other proteins in each molecular weight complex.

In order to define which proteins are associated with Kcc2 in the neuronal plasma membrane we developed a novel methodology for isolating stable multi-protein complexes enriched in this transporter. To do so, fresh isolated mouse forebrain was homogenized in detergent-free conditions to preserve organelle structure. The resulting homogenate was then subjected to differential gradient-based centrifugation and the plasma membrane fraction was isolated (Figure 1A). To confirm effective fractionation, we immunoblotted for established protein markers for each specific organelle (Figure 1B); HSP90 (cytosol), HSP60 (mitochondria), calreticulin (ER/Golgi), and n-cadherin (n-Cadh) (plasma membrane). Each organelle fraction showed enrichment for its respective marker protein. Importantly, the plasma membrane fraction was both enriched for n-Cadh and depleted for HSP60 compared to the total lysate, demonstrating efficient separation of plasma membrane and mitochondria, a common contaminant of membranous fractions. Kcc2 was enriched in the ER fraction and highly enriched in the mitochondrial and plasma membrane fractions. The enrichment of Kcc2 in our target fraction, the plasma membrane fraction, was highly encouraging for downstream purification. The presence of Kcc2 in the mitochondrial fraction could be an interesting future area of research, as chloride homeostasis is fundamental for correct mitochondrial function.

Following detergent solubilization, membrane fractions were subjected to immunoprecipitation with a monoclonal antibody directed against the intracellular C-terminus of Kcc2 covalently coupled to protein G-coated ferric beads. Initial optimization experiments revealed that we were able to isolate approximately 65% of the Kcc2 available in the lysate and the lysate preparation produced minimal Kcc2 oligomerization when resolved by SDS-PAGE (Supplementary Figures S1A,B). Purified material was eluted under native conditions using a “soft elution buffer” containing 2% Tween-20 (Antrobus and Borner, 2011), and subjected to blue-native-PAGE (BN-PAGE; Figure 1C). We observed well-resolved bands by colloidal Coomassie staining at 300, 600, and 800 kDa. These bands were not seen in control experiments performed with non-immune mouse IgG. An immunoblot of the same sample confirmed that these bands contained Kcc2. Immunoblots of plasma membrane lysates separated by BN-PAGE showed that these distinct Kcc2 complex bands exist in the lysate and are not an artifact of or degraded by purification. Despite the high stringency of our method (with high speed centrifugation and low concentration SDS exposure), no monomeric Kcc2 was observed (∼150 kDa when resolved by SDS-PAGE), indicating that Kcc2 was maintained in higher order complexes. This method may disrupt low affinity, or transient protein interactions and favor the detection of higher affinity binding partners.

After we confirmed that these well-resolved bands contained Kcc2, we assessed their composition by LC-MS/MS following trypsin digestion. The proteomic approach we used is summarized in Figure 1D. First, we extracted peptide counts for quality control measurements (Figure 1E). The 300, 600, and 800 kDa bands contained an average of 443, 159, and 45 total peptides for Kcc2, respectively, which equated to average coverage of 44%, 35%, and 23% of the total Kcc2 protein sequence, respectively. In all three bands, the majority of peptides corresponded to the cytoplasmic and extracellular domains of Kcc2, while peptides corresponding to the transmembrane domains were rarely detected. The failure to detect these regions is consistent with their high hydrophobicity and subsequent low recovery by LC-MS/MS (Supplementary Figure S2A). To assess the complexity of each band we compared the average total peptide counts for Kcc2, with the average total peptide counts for all proteins that were detected in our LC-MS/MS spectra (Figure 1F). In the 300, 600, and 800 kDa bands, Kcc2 was the most abundant protein detected, however, the proportion of peptides for Kcc2 relative to peptides for all other detected proteins decreased with increasing complex molecular mass, this is likely due to the increased protein complexity of the high molecular weight complexes. Taken together, these results demonstrate our experimental methodology facilitates the isolation of stable high molecular mass protein complexes enriched in Kcc2.

### Analyzing the Composition of Stable Protein Complexes of Kcc2

Having confirmed the veracity of our Kcc2 purifications we investigated the reproducibility of the proteins associated with Kcc2 in each of the bands. First, we extracted the spectral index normalized to global intensity (SI_*GI*_), a label-free quantitative measurement for each detected protein, using the MSnbase package in R (Supplementary Tables S2–S14). We compiled a data matrix of all the proteins and SI_*GI*_ values detected by proteomic analysis in each of our samples, including those detected as binding to non-immune IgG control beads. We then performed a Welch *t*-test to identify proteins significantly enriched in the Kcc2 IP compared to the control. This resulted in the identification of 246 proteins in total across all molecular weight complexes.

**FIGURE 2:**
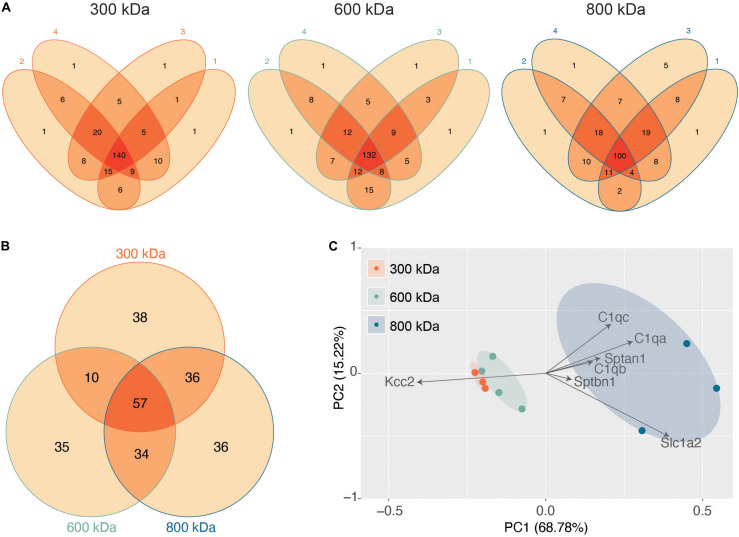
Stable protein complexes contain large amounts of Kcc2 and a robust set of associated proteins, which differ subtly between the different molecular weight bands. **(A)** Venn diagrams showing the number and overlap of significantly enriched Kcc2-associated proteins identified in each of 4 biological replicates in each molecular weight complex. **(B)** Venn diagram showing the overlap in significantly enriched associated proteins detected in all 4 replicates for each molecular weight complex. **(C)** PCA analysis of each biological replicate for each molecular weight complex based on their protein composition. PCA loadings were also included to show the contribution of each protein to the position of sample on the PCA plot.

Next, we evaluated the reproducibility of the significantly detected proteins in each of the 4 biological repeats for each band (Figure 2A). The vast majority of proteins detected were present in at least three of the 4 biological replicates, illustrating the reproducibility of our purifications and analyses. Only proteins detected in all 4 repeats were taken forward for further analysis as “significantly enriched proteins” (Supplementary Table S1). We then compared these “significantly enriched proteins” between the 300, 600, and 800 kDa complexes (Figure 2B). The 300 kDa band contained many unique proteins not identified in the 600 or 800 kDa bands. These may be large proteins that had dissociated from Kcc2 complexes. However, the amounts of these proteins were an order of magnitude lower than Kcc2 itself, indicating that the 300 kDa band is mostly Kcc2 alone. The 600 and 800 kDa bands contained proteins in more comparable quantities to Kcc2 itself, indicating these are likely to be Kcc2-binding proteins.

Finally, we used principal component analysis (PCA) to assess the degree of similarity in binding protein patterns between all 12 of the proteomic samples (Figure 2C). PCA identifies variance between samples and expresses the degree of similarity by proximity in a PCA plot. We did this to both assess the reproducibility of the biological replicates and the degree of difference in the binding proteins for each complex. The “significantly enriched proteins” were assembled into a matrix of all repeats and detected proteins along with SI_*GI*_ values for each. The data were normalized by z-transformation and the PCA plot created using the default settings in the ggfortify package (accessed January 2019) in R (Hilario and Kalousis, 2008). The datasets clearly separated into biological repeats of the different mass bands. PC1, which accounted for 68.78% of the variance, was highly positively correlated with amount of Sptan1, Sptbn1, and C1qa/b/c, and negatively correlated with the amount of Kcc2. This is consistent with the detection of more spectrin and C1q in the higher molecular weight bands, and a concomitant decrease in the amount of detected Kcc2 due to higher sample complexity. PC2, which accounted for 18.53% of the variance was also positively correlated with the detection of more C1qa/b/c. This demonstrates that we have identified a reproducible set of binding proteins for the 300, 600, and 800 kDa bands. While there is a substantial degree of overlap between the interacting proteins from each molecular mass band (Figure 2B), their protein content is different enough that they can be clearly differentiated by PCA.

**FIGURE 3:**
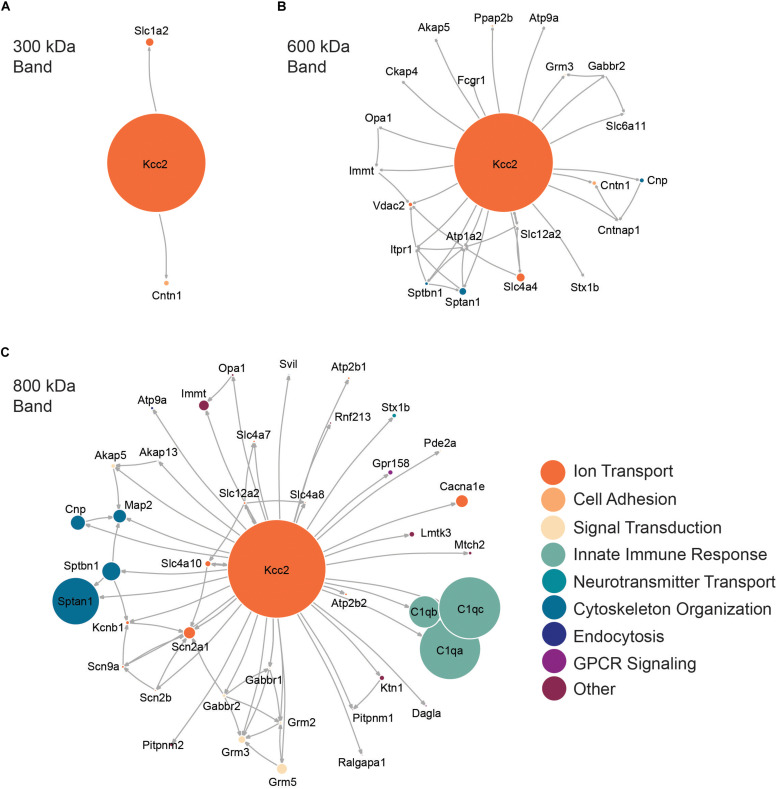
High molecular weight complexes of Kcc2 contain a robust set of proteins from multiple functional classes, with some highly interconnected subnetworks. Protein network diagrams for **(A)** the 300 kDa band, **(B)** the 600 kDa band, and **(C)** the 800 kDa band. Only proteins with >2% of the SI_GI_ values for Kcc2 were included to remove “trace” binding proteins. Known interactions were obtained using stringent high confidence, direct experimental association parameters from STRINGdb. These were used to construct a network diagram of protein nodes and arrows to indicate known interactions. The interactions for each protein with Kcc2 were included as discovered here. The node diameter was scaled relative to the SI_*GI*_ values detected for each protein. An overlay of Gene Ontology (GO) Biological Process terms was used to provide protein classification information (*n* = 4).

Next, we performed network analysis on the “significantly enriched proteins.” We omitted proteins whose SI_*GI*_ values were lower than 2% of those detected for Kcc2, to remove “trace” binding proteins. Network analysis was carried out using data from STRINGdb for known experimental interactions (Szklarczyk et al., 2019) as previously described (Goldman et al., 2019). We overlaid the highest scoring Gene Ontology (GO) Biological Process term for each protein to provide insight into protein function (Szklarczyk et al., 2019). We also scaled the node size representing each protein to the SI_*GI*_ values detected for each protein. In this way we were able to visualize quantitative Kcc2 networks and subnetworks along with developing insights into the functional groups of proteins that associate with Kcc2 (Figures 3A–C). In the 300 kDa band, Kcc2 (SI_*GI*_ of 0.184) was detected at more than 10-fold higher abundance than the next most abundant proteins; Slc1a2 (0.0159) and Cntn1 (0.0037). The 600 kDa protein complex contained high levels of Kcc2 (0.0792). A subnetwork of Sptan1 (0.0062) and Sptbn1 (0.0044) also emerged, along with Slc4a4 (0.0072). The associated proteins were largely involved in cytoskeleton organization or ion transport according to the top scoring Biological Process Gene Ontology terms. The 800 kDa complex also contained high amounts of Kcc2 (0.0229) along with comparable amounts of Sptan1 (0.0110), Sptbn1 (0.0044), C1qa/b/c (0.0142, 0.0075, 0.0144). There were three main subnetworks containing C1qa/b/c, another containing Sptan1/Sptbn1/Cnp/Map2, and another containing Grm2/3/5/Gabbr1/2). The 800 kDa complex contained more functionally diverse proteins involved in the innate immune response, GPCR signaling, and signal transduction, along with cytoskeleton organization and ion transport as seen in the 600 kDa band.

### Confirming the Association of “Robust Binding Proteins” With Kcc2

**FIGURE 4:**
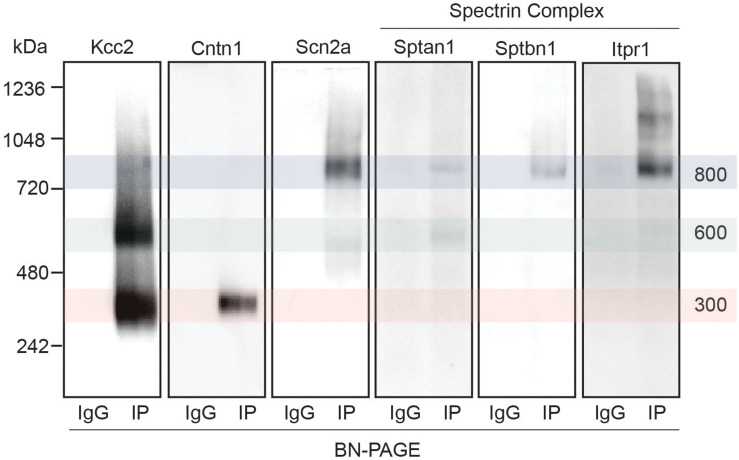
Immunoblots confirm the presence of several interactors in protein complexes that contain Kcc2. Kcc2 protein complexes were isolated from forebrain plasma membrane fractions of 8–12-week-old mice by immunoprecipitation, resolved by BN-PAGE and immunoblotted for components of the spectrin subnetwork. IPs were loaded adjacent to parallel IgG reactions to demonstrate specificity. Cntn1 and Scn2a were included as a further demonstration of band specificity, as proteomic analysis suggested specific enrichment in the 300 kDa and 800 kDa bands, respectively. Transparent overlays mark the location of each Kcc2 complex.

Next, we set out to confirm the association of a subset of the newly identified interactors with Kcc2. We chose to focus on the spectrin complex as it was highly prominent and contained structural, regulatory, and other ion transporting proteins. We immunoblotted isolated Kcc2 protein complexes resolved by BN-PAGE for Sptan1, Sptbn1, Itpr1, and Scn2a (Figure 4). We also included Cntn1 and Scn2a as specificity controls as they were particularly abundant in the low (300 kDa) and high (800 kDa) bands, respectively according to the proteomic analyses. No immunoreactivity was observed in the IgG control lanes. Mirroring the proteomic data Cntn1 showed specificity for the 300 kDa band, whereas Scn2a showed strong specificity for the 800 kDa band. In agreement with the proteomic data, none of the components of the spectrin subnetwork were present in the 300 kDa band. Sptan1 was present in the 600 kDa band and was also present in the 800 kDa band along with Sptbn1 and Itpr1.

### Kcc2 Co-localizes With the Spectrin Complex on, or Close to the Neuronal Plasma Membrane

**FIGURE 5:**
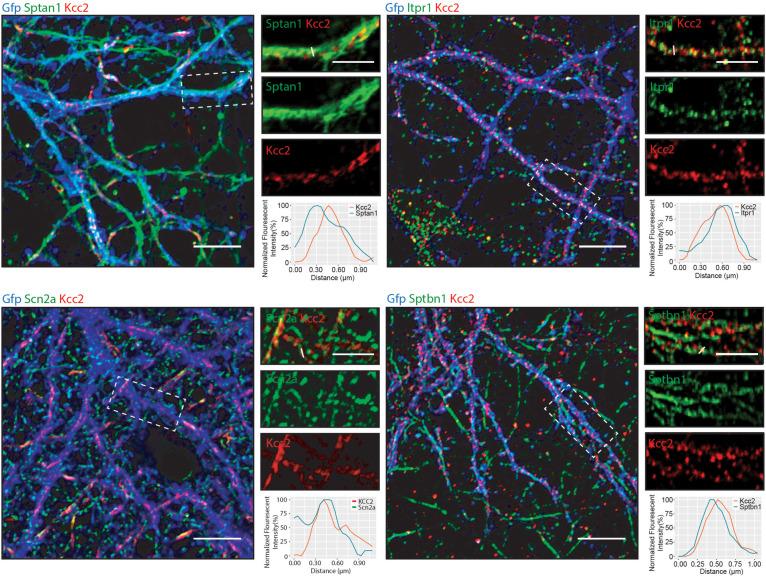
Components of the spectrin subnetwork co-localize with Kcc2 in several neuronal cellular compartments. Primary cultured neurons from P0 pups were infected with CamKII-AAV-GFP at DIV 3 (to visualize cell morphology and to identify excitatory neurons) and fixed at DIV 21. The cells were immunostained for Kcc2 and components of the spectrin subnetwork; Sptan1, Sptbn1, Itpr1, and Scn2a. Co-localization was measured using line scans (white lines) and represent the normalized fluorescent intensity of single pixels (approximately 1 μm) on dendrites (*n* = 3). Scale bars are 10 μm for large images and 5 μm for cropped images.

Having established that Kcc2 forms protein complexes with components of the spectrin complex, we used immunocytochemistry to investigate the proximity of each to Kcc2. We used DIV 21 mouse primary cultured cortical/hippocampal neurons infected with CamKII-AAV-GFP to ensure we imaged excitatory neurons and to visualize the morphology of the cell and identify whether the interaction between Kcc2 and the spectrin complex was restricted to specific cellular compartments (Figure 5). Sptan1 exhibited widespread staining along dendrites, which co-localized with puncta of Kcc2. Itpr1 exhibited punctate staining with convincing co-localization with Kcc2 in the dendritic compartment. Sptbn1 was present in dendritic clusters with Kcc2 mostly located on the edges. Scn2a, outside of the easily identifiable AIS regions, was also robustly co-localized with Kcc2. These results were also reflected in density plots carried out as previously described (Bolte and Cordelières, 2006; Davenport et al., 2017). Taken together, these data demonstrate that proteins associated in high molecular weight complexes with Kcc2 are also highly co-localized with Kcc2 in neurons.

### Kcc2 Is at or in Close Proximity to Inhibitory Synapses

**FIGURE 6:**
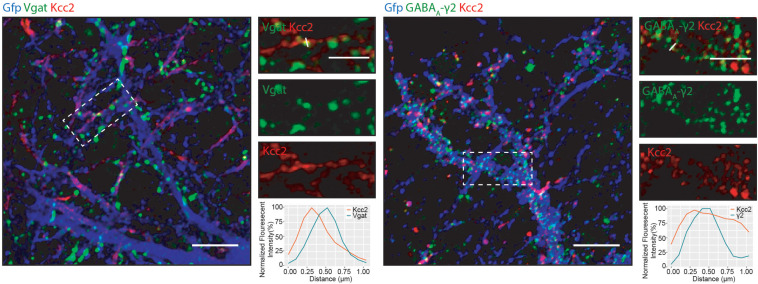
Kcc2 is localized at inhibitory synapses. Primary cultured neurons from P0 pups were infected with CamKII AAV-GFP at DIV 3 and fixed at DIV 21. The cells were immunostained for Kcc2 and the inhibitory pre- and post-synaptic markers Vgat and GABA_A_-γ2. Co-localization was measured using line scans (white lines) and represent the normalized fluorescent intensity of single pixels (approximately 1 μm) on dendrites (*n* = 3). Scale bars are 10 μm for large images and 5 μm for cropped images.

We detected several proteins in complex with Kcc2 that have been proposed to be located at inhibitory synapses. To investigate this further, we used immunocytochemistry to visualize Kcc2 relative to the inhibitory pre- and post-synaptic marker proteins Vgat and GABA_A_-γ2, respectively (Figure 6). Kcc2 puncta appeared to be immediately adjacent to Vgat puncta, and co-localized robustly with GABA_A_-γ2, suggesting Kcc2 could be in close proximity to the post-synaptic region of inhibitory synapses.

### Kcc2 Is Highly Phosphorylated at Multiple Sites in the Murine Plasma Membrane

**FIGURE 7:**
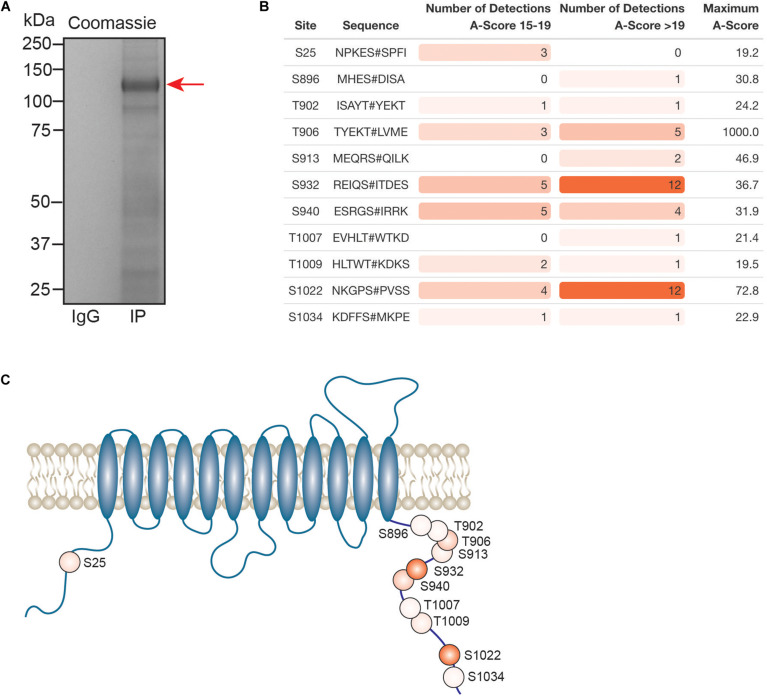
In the neuronal plasma membrane, Kcc2 can be phosphorylated at 11 distinct sites. **(A)** Kcc2 was isolated from plasma membrane fractions of mouse forebrain, resolved by SDS-PAGE and visualized using colloidal Coomassie. **(B)** Table of detected high confidence Kcc2 phosphorylation sites. Site numbering is according to the mature UniProt Kcc2 protein sequence. A-score were determined as previously described (Beausoleil et al., 2006). A score 15–19 and >19 represent a >90% and >99% confidence score, respectively. The number of times a particular phospho-peptide was detected at either A-score range was recorded across 4 individual experiments, along with the maximum recorded A-score (*n* = 4). Table cells are color coded according to the number of detections. **(C)** Illustrative diagram of high confidence Kcc2 phosphorylation site positions color coded according to the number of detections at an A-score >19. Transmembrane domain positions according to the UniProt database.

Kcc2 expression levels, membrane trafficking and activity have been shown to be subject to modulation via multiple phosphorylation sites within the C-terminal cytoplasmic domain. As we had succeeded in obtaining highly purified Kcc2 from murine brain plasma membranes, we also analyzed Kcc2 using phosphoproteomics. To do so, we immunoprecipitated Kcc2 from plasma membrane fractions prepared from WT mice as outlined in Figure 1. To limit dephosphorylation purified material was resolved by SDS-PAGE and visualized using Coomassie (Figure 7A). Major bands of 125 kDa were seen with material purified on the Kcc2 antibody, but not control IgG. The band was excised, proteins were digested with trypsin, and analyzed by LC-MS/MS. LC-MS/MS confirmed the 125 kDa band was highly enriched for Kcc2 with an average of 884 peptides detected (*n* = 4), which equates to coverage of 53% (Supplementary Figure S2).

We identified 11 high confidence Kcc2 phosphorylation sites based on A-scores either 15–19 or greater than 19, which equates to 90% or 99% confidence in assignment, respectively (Figure 7B and Supplementary Table S15). One site (S25/26), is located on the N-terminus of Kcc2, whereas the other 10 sites are located on the C-terminus (S896, T902, T906, S913, T932, S940, T1007, T1009, S1022, and S1034) (Figure 7C). The number of times a phosphopeptide was detected with an A-score 15–19 or >19 was recorded across 4 biological replicates, to provide a measure of abundance and confidence in each phosphorylation site. We detected the three well characterized sites; T906, S940, and T1007, however, T1007 was only detected once with a qualifying A-score. The two most regularly detected high confidence phosphosites were S932, and S1022 (detected 18 and 16 times, respectively), which are relatively unstudied phosphosites. T906 and S940 were also detected with high confidence several times. Collectively, these results demonstrate that plasma membrane Kcc2 is phosphorylated at multiple sites at the N- and C-termini, at both known and novel sites (Figure 7C).

## Discussion

### Isolation of Stable Multi-Protein Complexes Enriched in Kcc2

The K^+^/Cl^–^ co-transporter, Kcc2, is expressed at the cell surface of mature neurons where it is of fundamental importance for maintaining intracellular chloride levels, and thereby the efficacy of neuronal inhibition. To gain insights into how neurons regulate the plasma membrane accumulation and activity of Kcc2, we applied a method for the isolation of native multi-protein complexes enriched in this transporter from brain plasma membranes (Suski et al., 2014; Agez et al., 2017). Following plasma membrane isolation by differential centrifugation, immuno-affinity purification, BN-PAGE/SDS-PAGE and LC-MS/MS we were able to isolate highly purified preparations of Kcc2. When resolved by SDS-PAGE we obtained a single band at approximately 140 kDa. When resolved by BN-PAGE we observed major molecular mass species of 300, 600, and 800 kDa. This methodology provided very high sequence coverage of Kcc2 (approximately 45% of the total sequence, and 90% of the intra- and extracellular soluble regions), maximizing the probability of detecting associated proteins and post-translational modifications. It could therefore prove to be a beneficial methodology for the isolation of other plasma membrane proteins from brain tissue, that not only maintains protein complexes, but also post-translational modifications.

### Kcc2 Exists as Dimers in the Neuronal Plasma Membrane

Kcc2 consists of 1139 amino acids including the signal sequence, which equates to a mass of 126 kDa without post-translational modifications. Kcc2 is, however, subject to extensive post-translational modification including glycosylation at 6 sites so is often observed in a band of around 140 kDa (Agez et al., 2017) when resolved by SDS-PAGE. Previous work has shown extensive aggregation of Kcc2 following SDS-PAGE that results in a band at 250 kDa, however, this depends on whether it is heterologously expressed or the manner in which it is prepared from neuronal tissues (Medina et al., 2014). Indeed, we see no aggregation in our samples following SDS-PAGE, possibly due to the use of low detergent concentrations and samples immediately processed for SDS-PAGE without freeze thaw cycles (Supplementary Figure S1). Interestingly, when resolved by BN-PAGE, Kcc2 protein complexes form distinct bands at 300 kDa, 600 kDa, and 800 kDa. The 300 kDa band contains approximately 10-fold more Kcc2 than the next most abundant protein, indicating that this band consists largely of Kcc2. At 300 kDa, this would indicate that in the plasma membrane Kcc2 exists as homodimers, which is consistent with recently published studies on recombinant Kcc2 molecules visualized using cryo-EM (Agez et al., 2017). This, however, is the first demonstration that endogenously expressed Kcc2 is in dimeric form in the murine brain.

### Kcc2 Forms Stable Multi-Protein Complexes in the Plasma Membrane

The most abundant protein in the 600 kDa and 800 kDa bands was consistently Kcc2, confirming that these are legitimate Kcc2-containing protein complexes. Significantly the ratio of the associated proteins relative to Kcc2 increased with molecular mass. Given the stringency of our methodology and the use of native conditions these species represent stable-protein complexes that primarily reside in the plasma membrane. The proteins detected by a previous proteomic analysis of Kcc2 complexes (Mahadevan et al., 2017) overlapped with 13% and 16% of the proteins we detected in the 600 kDa and 800 kDa bands, respectively. This low degree of overlap is likely due to the methodological differences between the studies; the previous work extracted Kcc2 complexes from a crude membrane preparation and used on-bead digest prior to LC-MS/MS, rather than complexes purified from a plasma membrane fraction resolved by BN-PAGE, then distinct bands excised for analysis. This difference in biochemical preparation is more likely to detect transient interactions due to less sample processing, but is also far less stringent, and results in a highly complex sample being introduced to the LC-MS/MS, which can significantly impact resolution. However, the method presented here ensured a sample of reduced complexity analyzed by LC-MS/MS, resulting in a higher resolution snapshot of the proteins associated with high affinity with Kcc2 on the neuronal plasma membrane, which were then measured using label-free quantification. Similarly to Mahadevan et al. (2017), we also did not detect Neto2 (Ivakine et al., 2013), Grik2 (Gluk2) (Mahadevan et al., 2014), Epb41l1 (4.1n) (Li et al., 2007), Arhgef7 (Beta-pix) (Chevy et al., 2015), Rcc1 (Garbarini and Delpire, 2008) or the signal transduction molecules; Pkc, Wnk, Spak or Osr (Lee et al., 2007; Friedel et al., 2015). This could be either due the transient or low affinity nature of these interactions, or that these interactors are less abundant in complex with Kcc2 than the interactors presented here, as this quantitative methodology provides information on associated proteins in rank order of abundance. We did, however, detect GABA_*B*_Rs which have previously shown to be associated with Kcc2 (Wright et al., 2017), further confirming the veracity of our purification strategy.

### Kcc2 Is Associated With Proteins Enriched at Excitatory and Inhibitory Synapses

Previous work has shown that Kcc2 is located at or in close proximity to excitatory synapses (Gulyás et al., 2001; Chamma et al., 2012; Mahadevan et al., 2017). In agreement with this we detected well characterized excitatory synapse proteins such as Cntn1 (Cijsouw et al., 2018), and Grm2/3 (Ting et al., 2016; Spence et al., 2019). Interestingly, we also identified several proteins associated with inhibitory synapses including Cacna1e (Nakamura et al., 2016), and Cyfip1 (Supplementary Table S1; Davenport et al., 2019). Grm5 has been detected in high abundance at both excitatory and inhibitory synapses (Nakamura et al., 2016; Spence et al., 2019). Immunostaining of Kcc2 and inhibitory synapse marker proteins presented here, shows that Kcc2 is located in close proximity to inhibitory synapses. Taken together, these data suggest that Kcc2 is not located exclusively at either type of synapse but is likely to be positioned at or in close proximity to both. This is consistent with the suggestion that Kcc2 may play a role in the regulation of excitatory and inhibitory synapses (Awad et al., 2016). It is also of note that many of the proteins we detected are protein products of autism- and epilepsy-risk genes.

### Kcc2 Is Associated With the Spectrin Cytoskeleton

The most prominent subnetwork that we identified associated with Kcc2 was the spectrin complex, which consisted of Sptan1, Sptbn1, Itpr1, and Map2 (Shi et al., 2009). Sptan1, the most abundant binding protein in the higher molecular weight complexes, is a structural protein that forms dimers with a β-spectrin isoform such as Sptbn1. These spectrin dimers interact with actin filaments and ankyrins, to regulate the expression of plasma membrane proteins, including calcium channels (including Cacna1e) (Garcia-Caballero et al., 2018), sodium channels (including Scn2a) (Bouzidi et al., 2002), potassium channels, and Itpr1 (El Refaey and Mohler, 2017). Cntn1 is also thought to interact with spectrins either directly or via Caspr1 (Querol et al., 2017). The importance of this complex is reflected in the debilitating diseases associated with spectrin mutations. Sptan1 mutation is associated with epilepsy (Tohyama et al., 2015), and Sptbn1 is implicated in ASDs (Kim H. G. et al., 2008). Both Scn2a and Itpr1 are high risk ASD genes identified by SFARI. Sptan1 cleavage by calpains, as also occurs with Kcc2 (Puskarjov et al., 2012), is an established marker of neuronal damage (Yan and Jeromin, 2012).

Our data suggests that Kcc2 may interact directly with Sptan1, as it is present in the 600 kDa band with Kcc2. In contrast, Sptbn1 and Itpr1 are likely to require the binding of Sptan1 to associate with Kcc2, as they are most highly abundant in the 800 kDa band, and absent in the 600 kDa band (Figure 4). This hypothesis is also consistent with the shift in molecular weight observed in the Kcc2 complex. A Kcc2 dimer of approximately 300 kDa would run at approximately 600 kDa with an associated Sptan1 protein (285 kDa). The subsequent association of either Sptbn1 (275 kDa), or Itpr1 (310 kDa) would shift the mass to around 800 kDa. Sptan1 has a variety of binding domains where interactions with Kcc2 could occur, such as SH3 domains (Ackermann and Brieger, 2019). It has previously been proposed that Kcc2 also contains a non-canonical SH3 domain in its C-terminus (Llano et al., 2015).

The association of Kcc2 with the spectrin complex would not only stabilize Kcc2 in the plasma membrane, but also provide a link to signaling processes via Itpr1. Itpr1 is present at PM/ER junctions and following activation by G-protein coupled receptors (GPCR), releases Ca^2+^ from the ER. High Ca^2+^ concentration would lead to localized PKC activation, which has been shown to phosphorylate Kcc2 at multiple sites (Lee et al., 2007). This may also be consistent with other GPCR signaling proteins detected here, such as GPR158. The presence of Kcc2 and its associated proteins at PM/ER contact sites is a potentially exciting observation that could influence Kcc2 synthesis and regulation and will be the subject of future studies. Finally, we also detected high quantities of C1qa/b/c in high molecular weight complexes with Kcc2. This is a potentially important observation, due to the proximity of Kcc2 to both excitatory and inhibitory synapses and the role of C1q proteins in synaptic elimination during development and in disease (Schafer and Stevens, 2010).

### Plasma Membrane Populations of Kcc2 Are Phosphorylated on Up to 11 Residues

We used the same purification techniques to analyze endogenous Kcc2 phosphorylation from the neuronal plasma membrane. Phosphorylation plays a key role in regulating the transporter activity. We detected 11 residues including the well characterized sites; T906, S940, and T1007. These play a critical role in regulating Kcc2 function (Moore et al., 2018; Pisella et al., 2019). Interestingly, T1007 was only detected above the accepted higher A-score threshold once. T1007 phosphorylation is inversely correlated with Kcc2 function (Kahle et al., 2013), and therefore this could suggest that T1007 is largely absent in PM-associated Kcc2, where it is active.

In addition to the well characterized sites we also detected 8 further phosphorylation sites. These included S25/S26 and S1022 consistent with recent publications (Weber et al., 2014; Agez et al., 2017). We also identified 5 further sites of phosphorylation; S896, T902, S913, S932, T1009, and S1034. S932 and S1022 were the most frequently detected phosphosites, indicating that Kcc2 in the PM is highly phosphorylated at these residues. S932 is in close proximity to the well characterized S940 site, which is vital for correct Kcc2 function (Silayeva et al., 2015). S1022 lies adjacent to, or within the isotonic domain of Kcc2, suggesting that its phosphorylation may impact its basal activity (Mercado et al., 2006). We isolated several proteins in stable complexes with Kcc2 that play a direct role in protein phosphorylation; including Lmtk3, Akap5, Akap13. Conversely, the differential phosphorylation of Kcc2 may influence the proteins it associates with, influencing the subcellular localization and turnover of Kcc2. The expanded evidence of associated kinases and novel phosphorylation sites presented here, will be the subject of future investigation.

In conclusion we have demonstrated a highly efficient method for isolating Kcc2 from murine plasma membranes, in a highly phosphorylated form within its native protein complex. This has allowed us to perform quantitative studies on stable Kcc2 protein complexes and phosphorylation in parallel from the same preparation. We confirm that endogenous Kcc2 is in homo-dimeric conformation in murine plasma membranes. These dimers associate with a spectrin subnetwork that is likely to not only stabilize it in the plasma membrane but provide an environment for regulating its phosphorylation at the 11 potential sites.

### Experimental Procedures

Unless otherwise stated chemicals were obtained from Sigma-Aldrich, St. Louis, MO, United States.

### Animals

Animal studies were performed according to protocols approved by the Institutional Animal Care and Use Committee of Tufts Medical Center. 8–12-week-old male and female mice were kept on a 12 h light/dark cycle with *ad libitum* access to food and water.

### Antibodies

The following antibodies were used for immunoprecipitation (IP), immunoblot (IB), or immunocytochemistry (Icc): Cntn1 (rabbit, IB, Abcam Ab66265), Itpr1 (rabbit, IB/Icc, Alomone Acc-019), Kcc2 (mouse, IP/Icc, NeuroMab 75-013), Kcc2 (rabbit, IB/Icc, Millipore 07-432), Scn2a (mouse, Icc, NeuroMab 75-024), Scn2a (rabbit, IB, Alomone ASC-002), Sptan1 (mouse, Icc, Abcam Ab11755), Sptan1 (rabbit, IB, Cell Signaling 21225), Sptbn1 (rabbit, IB/Icc, Abcam Ab72239), α-Tubulin (mouse, IB, Sigma T9026), vGAT (guinea pig, Icc, SYSY 131004), GABA γ2 (mouse, Icc, SYSY 224011), GFP (chicken, Icc, Abcam Ab13970).

### Immunoblotting

Sodium dodecyl sulfate polyacrylamide gel electrophoresis (SDS-PAGE) was carried out as previously described (Schiavon et al., 2018). Briefly, proteins were isolated in RIPA lysis buffer (50 mM Tris, 150 mM NaCl, 0.1% SDS, 0.5% sodium deoxycholate, and 1% Triton X-100, pH 7.4) supplemented with mini cOmplete protease inhibitor and PhosSTOP phosphatase inhibitor tablets. Protein concentration was measured using a Bradford assay (Bio-Rad, Hercules, CA, United States). Samples were diluted in 2x sample buffer and 20 μg of protein was loaded onto a 7–15% polyacrylamide gel depending on the molecular mass of the target protein. After separation by SDS-PAGE, proteins were transferred onto nitrocellulose membrane. Membranes were blocked in 5% milk in tris-buffered saline 0.1% Tween-20 (TBS-T) for 1 h, washed with TBS-T, and then probed with primary antibodies diluted in TBS-T (dilution and incubation time dependent on the antibody). The membranes were washed and incubated for 1 h at room temperature with HRP-conjugated secondary antibodies (1:5000 – Jackson ImmunoResearch Laboratories, West Grove, PA, United States). Protein bands were visualized with Pierce ECL (Thermo Fisher Scientific) and imaged using a ChemiDoc MP (Bio-Rad). Band intensity was compared to α-tubulin as a loading control.

### BN-PAGE

For blue-native polyacrylamide gel electrophoresis (BN-PAGE), proteins were isolated using Triton lysis buffer (150 mM NaCl, 10 mM Tris, 0.5% Triton X-100, pH 7.5). Samples were diluted in 4x NativePAGE sample buffer and G250 additive. Samples were loaded onto 4–16% NativePAGE gradient gels and run using a mini gel tank (Thermo Fisher Scientific, Waltham, MA, United States). Gels were run for approximately 2 h and then prepared for Coomassie staining or immunoblotting. For Coomassie staining the gels were fixed in 50% ethanol and 10% acetic acid, washed in 30% ethanol, washed in water, then stained with EZ blue stain. The gels were destained in ultrapure water, imaged using a ChemiDoc MP (Bio-Rad), and bands were excised for liquid chromatography tandem mass spectrometry (LC-MS/MS). For immunoblotting, proteins were transferred to PVDF membrane overnight. The membranes were then fixed in 8% acetic acid, washed with ultrapure water and air-dried before being briefly destained with 100% methanol. The membranes were then blocked, immunoblotted, and imaged as described above.

### Plasma Membrane Isolation

Plasma membrane isolation from mouse forebrain was carried out using a modified version of a previously described method (Suski et al., 2014). Briefly, mouse forebrain was rapidly isolated and placed in ice-cold starting buffer [225 mM mannitol, 75 mM sucrose, 30 mM Tris–HCl (pH 7.4)]. The tissue was then transferred to ice-cold isolation buffer (225 mM mannitol, 75 mM sucrose, 0.5% (wt/vol) BSA, 0.5 mM EGTA, 30 mM Tris–HCl, pH 7.4) supplemented with mini cOmplete protease inhibitor and PhosSTOP. Forebrain tissue from 5 to 7 mice was pooled and used for each Kcc2 immunoprecipitation biological replicate. The brains were homogenized in the isolation buffer using 14 strokes of a Dounce homogenizer. The samples were transferred to centrifuge tubes for centrifugation, which was carried out at 4°C throughout. The samples were initially spun at 800 × *g* for 5 min to remove nuclei and non-lysed cells. The pellet was discarded, and the supernatant was spun again at 800 × *g* for 5 min to remove residual nuclei and non-lysed cells. The supernatant was transferred to high speed centrifuge tubes and spun at 10000 × *g* for 10 min to remove mitochondria. The pellet was discarded, and the supernatant spun again at 10000 × *g* for 10 min to remove mitochondrial contamination. The supernatant was then spun at 25000 × *g* for 20 min to pellet plasma membranes. The pellet was resuspended in starting buffer and spun again at 25000 × *g* for 20 min to remove cytosolic and ER/Golgi contamination. Finally, the plasma membrane fraction was purified on a discontinuous sucrose gradient (53%, 43%, 38% sucrose), and spun at 93000 × *g* for 50 min to remove residual contamination from cytosol and other membranous compartments.

### Primary Neuron Culture

Mouse cortical and hippocampal mixed cultures were created from P0 mouse pups as previously described (Kelley et al., 2018). Briefly, P0 mice were anesthetized on ice and the brains removed. The brains were dissected in Hank’s buffered salt solution (Invitrogen/Thermo Fisher Scientific) with 10 mM HEPES. The cortices and hippocampi were trypsinized and triturated to dissociate the neurons. Cells were counted using a hemocytometer and plated on poly-l-lysine-coated 13 mm coverslips in 24-well plate wells at a density of 2 × 10^5^ cells/ml in Neurobasal media (Invitrogen/Thermo Fisher Scientific). At days *in vitro* (DIV) 3, 10^5^ genomic copies per cell of CamKII-AAV9-GFP (Addgene, Watertown, MA, United States) were added to the neuronal media. After 24 h, the media was replaced with new conditioned media. At DIV 21, cells were fixed in 4% paraformaldehyde in PBS for 10 min at room temperature. They were then placed in PBS at 4°C until being processed for immunocytochemistry.

### Immunocytochemistry

Fixed primary neurons were permeabilized for 1 h in blocking solution (3% bovine serum albumin (BSA), 10% normal goat serum, 0.2 M glycine in phosphate buffered saline (PBS) with 0.1% Triton-X100). Cells were exposed to primary and then fluorophore-conjugated secondary antibodies (Alexa Fluor 488, 555, and 647; Thermo Fisher Scientific, 405 Dylight; Jackson ImmunoResearch) diluted in blocking solution for 1 h each at room temperature. The coverslips were then washed in PBS, dried, and mounted onto microscope slides with Fluoromount-G (SouthernBiotech, Birmingham, AL, United States). The samples were imaged using a Nikon Eclipse Ti (Nikon Instruments, Melville, NY, United States) or Leica Falcon (Leica Microsystems, Buffalo Grove, IL, United States) confocal microscope using a 60x oil immersion objective lens. Image settings were manually assigned for each fluorescent channel. For image processing, the background was subtracted for each fluorescent channel and the median filter was applied (Radius = 1 pixel) on Fiji Software (Schindelin et al., 2012). The line scans (white lines) used for protein localization were generated using the plotProfile function in Fiji and represent the fluorescent intensity of single pixels against the distance of a manually drawn line (approximately 1 μm) on dendrites as previously described (Bolte and Cordelières, 2006; Davenport et al., 2017).

### Densitometry

For Western blot analysis, bands from raw images were analyzed using the Fiji densitometry features. Biological replicates were run on the same gels for comparison, and area under the curve was calculated for each band. Average signal and standard error were calculated for each treatment group and ANOVA carried out using R for statistical comparison of protein expression levels.

### Protein Purification

Protein G Dynabeads (Thermo Fisher Scientific) were washed three times with phosphate buffered saline with 0.05% Tween-20 (PBS-Tween). The beads were resuspended in PBS-Tween and incubated overnight at 4°C with antibodies for the target protein at an experimentally predetermined bead:antibody ratio (Supplementary Figure S1). The antibody was crosslinked onto the beads by washing twice with 0.2 M triethanolamine (pH 8.2) (TEA), and then incubated for 30 min with 40 mM dimethyl pimelimidate (DMP) in TEA at room temperature. The beads were transferred to 50 mM Tris (pH 7.5) and incubated at room temperature for 15 min. The beads were then washed three times with PBS-Tween and resuspended in solubilized plasma membranes in ice-cold Triton lysis buffer, supplemented with mini cOmplete protease inhibitor and PhosSTOP. The immunoprecipitation reaction was incubated overnight at 4°C. The beads were then washed three times with PBS-Tween and eluted either with 2x sample buffer (for SDS-PAGE) or soft elution buffer [0.2% (wt/vol) SDS, 0.1% Tween-20, 50 mM Tris–HCl, pH = 8.0] (Antrobus and Borner, 2011) (for BN-PAGE).

### Protein Analysis by LC-MS/MS

Excised gel bands were cut into 1 mm^3^ pieces and subjected to modified in-gel trypsin digestion, as previously described (Shevchenko et al., 1996). Briefly, gel pieces were washed and dehydrated with acetonitrile for 10 min and then completely dried in a speed-vac. Gel pieces were rehydrated with 50 mM ammonium bicarbonate solution containing 12.5 ng/μl modified sequencing-grade trypsin (Promega, Madison, WI, United States) and incubated for 45 min at 4°C. The excess trypsin solution was removed and replaced with 50 mM ammonium bicarbonate solution. Samples were then incubated at 37°C overnight. Peptides were extracted by washing with 50% acetonitrile and 1% formic acid. The extracts were then dried in a speed-vac (∼1 h). The samples were stored at 4°C until analysis. Before analysis the samples were reconstituted in 5–10 μl of HPLC solvent A (2.5% acetonitrile, 0.1% formic acid). A nano-scale reverse-phase HPLC capillary column was created by packing 2.6 μm C18 spherical silica beads into a fused silica capillary (100 μm inner diameter × ∼30 cm length) with a flame-drawn tip (Peng and Gygi, 2001). After equilibrating the column each sample was loaded via a Famos auto sampler (LC Packings, San Francisco, CA, United States) onto the column. A gradient was formed between solvent A (97.5% water, 2.5% acetonitrile, and 0.1% formic acid), and increasing concentrations of solvent B (97.5% acetonitrile, 2.5% water, and 0.1% formic acid). Acquisition time was 16–79 min. As each peptide was eluted, they were subjected to Nanospray ionization (NSI) and then entered into an LTQ Orbitrap Velos Pro ion-trap mass spectrometer (Thermo Finnigan, San Jose, CA, United States). MS1 was set at 70 k, with a scan range of m/z 85–2000, charge-state screening parameters +2 to +5, using a centroid acquisition mode, with a precursor ion isolation window of 2 m/z and 35% normalized collision energy. Eluting peptides were detected and the most intense were isolated using the Top 10 scan mode and fragmented by Higher-energy C-trap dissociation (HCD). MS2 ions were analyzed by an Orbitrap mass spectrometer with a resolution of 17.5 k and the dynamic exclusion settings; 30 s repeat duration, 60 s exclusion duration, *n* = 1, 10 ppm mass width, to produce a tandem mass spectrum of specific fragment ions for each peptide.

### Bioinformatics and Statistics

#### Peptide/Protein Searches

Peptide sequences (and hence protein identity) were determined by matching protein or translated nucleotide databases with the acquired fragmentation pattern using the MSGF+ (Kim and Pevzner, 2014). Raw mzXML files were used to search the UniProt mouse reference proteome (last modified May 4th 2020, containing 21989 sequences) also containing the Thermo list of common contaminants. The search was carried out using settings for high-resolution Orbitrap mass spectrometers, tryptic digestion, no limit to enzyme missed cleavages, 20 ppm precursor mass tolerance, charge states of +2 to +5, minimum and maximum peptide lengths of 6–40 amino acids in length, respectively, and a fixed modification of standard amino acids with C + 57. For phosphopeptide detection a variable modification of 79.9663 mass units to serine, threonine, and tyrosine was included in the database searches. Phosphorylation assignments were determined by the A score algorithm (Beausoleil et al., 2006). Peptide identification was scored by MSGF+ Q- (PSM-level target-decoy approach) and E- (expected number of peptides in a random database) scores. These were used for quality control in the initial protein screening process for associated proteins, and phospho-modified peptide screening (Kim S. et al., 2008).

#### Proteomic Data Analysis

The resulting mzID files from the spectral searches were combined with mzXML files using the MSnbase package in R (accessed July 20th 2020), and used to calculate peptide counts and the quantitative measurement; spectral index normalized to global intensity (SI_*GI*_). This has previously been shown to be an effective measurement for label-free protein quantification and to normalize replicate data well (Griffin et al., 2010). The mass spectrometry proteomics data have been deposited to the ProteomeXchange Consortium via the PRIDE (Perez-Riverol et al., 2019) partner repository with the dataset identifier PXD021368 and 10.6019/PXD021368.

For initial quality control we assessed protein coverage by aligning detected peptides for Kcc2 to the mouse Kcc2 reference sequence (UniProt ID: Q91V14) using the Multiple Sequence Alignment (MSA) package in R (accessed January 10th, 2019). We also recorded the number of peptides detected for Kcc2 and other detected proteins. Proteins with fewer than two peptides detected were removed for this analysis.

SI_*GI*_ values for each protein detected by Kcc2 immunoprecipitation were compared to non-immune IgG purifications by Welch *t*-test to identify significantly enriched proteins. Venn diagrams were produced using the Vennerable package in R (accessed January 10th, 2019), and only significantly detected proteins in all repeats were considered for downstream analysis. The SI_*GI*_ values for proteins contained within each gel band (Supplementary Table S1) were normalized by z-transformation (Supplementary Table S2) and used for principal Component Analysis (PCA), which was carried out using PCA functions in the ggfortify package in R (accessed January 10th, 2019). The significantly detected protein lists for each band were ordered according to SI_*GI*_ values and proteins with <50-fold lower SI_*GI*_ values than those for Kcc2 were removed as they were considered “trace” binding partners. The protein lists were compared against the latest version of the STRINGdb database (Szklarczyk et al., 2019) to establish known interactions and annotations for each protein using only high confidence, experimental evidence. Functional protein information, specifically the highest scoring Biological Process Gene Ontology terms, were extracted for each protein using the “mygene” package in R (accessed July 29th, 2020). The interaction for each protein with Kcc2 was imputed and network diagrams were constructed in R using the igraph package and the nodes were scaled to the SI_*GI*_ values for each protein (accessed February 1st, 2019).

## Data Availability Statement

The datasets presented in this study can be found in online repositories. The names of the repository/repositories and accession number(s) can be found in the article/Supplementary Material.

## Ethics Statement

The animal study was reviewed and approved by Tufts University.

## Author Contributions

JS and SM conceptualized the project, developed and analyzed data, and wrote the manuscript. GK performed immunocytochemistry experiments. CC and QR performed western blot experiments. MS and CB maintained the mouse colony and performed genotyping. PD, TD, NB, KA, and DA edited the manuscript. All authors contributed to the article and approved the submitted version.

## Conflict of Interest

SM serves as a consultant for AstraZeneca, and SAGE Therapeutics, relationships that are regulated by the Tufts University. SM holds stock in SAGE Therapeutics. The remaining authors declare that the research was conducted in the absence of any commercial or financial relationships that could be construed as a potential conflict of interest.

## Supplementary Material

FIGURE S1 Initial optimization of antibody:bead ratios for optimal Kcc2 enrichment by immunoprecipitation. Immunoblots were carried out on protein resolved by SDS-PAGE. Total lysate (TL) levels of Kcc2 were compared with immunoprecipitated (IP) samples purified with 0–10 μl of Kcc2 antibody crosslinked to 50 μl of Protein G Dynabeads, and the respective flow-through (FT) samples (to detect the amount of Kcc2 extracted from the total pool). **(B)** Densitometry of the immunoblot demonstrating considerable enrichment of Kcc2 by immunoprecipitation, and depletion of approximately 65% of the total Kcc2 available at the highest antibody:bead ratio.

FIGURE S2 The total Kcc2 peptides detected by LC-MS/MS across all biological repeats were aligned to the Kcc2 reference sequence (UniProt ID: Q91V14) to visualize protein coverage. **(A)** The Kcc2 coverage obtained from the 300, 600, and 800 kDa bands of Kcc2 resolved by BN-PAGE. **(B)** The Kcc2 coverage of wild type (WT) mouse samples from the ∼140 kDa band resolved by SDS-PAGE.

TABLE S1 A shortened list containing only significantly enriched proteins used for downstream analysis. SI_*GI*_ scores were used to detect significantly enriched proteins associated with Kcc2 by comparing them with control (IgG) purifications using a Welch *t*-test (Data Table 1).

TABLES S2–S14 A list of all the proteins detected by proteomic analysis in all samples with accompanying SI_*GI*_ values for each sample replicate. SI_*GI*_ scores were calculated from raw spectra data for all proteins detected across all samples. 300 kDa 1 – Data Table 2, 300 kDa 2 – Data Table 3, 300 kDa 3 – Data Table 4, 300 kDa 4 – Data Table 5, 600 kDa 1 – Data Table 6, 600 kDa 2 – Data Table 7, 600 kDa 3 – Data Table 8, 600 kDa 4 – Data Table 9, 800 kDa 1 – Data Table 10, 800 kDa 2 – Data Table 11, 800 kDa 3 – Data Table 12, 800 kDa 4 – Data Table 13, Control 1–4, Data Table 14.

TABLE S15 A list of the phosphorylated Kcc2 peptides detected. A summary table of the phosphorylated Kcc2 peptides detected. Calculated A-scores are displayed for each repeat as well as the highest MSGF + raw scores (peptide-spectrum match), *de novo* scores (optimal scoring peptide for the spectrum), spectral *E*-values, database *E*-values, and PSM-level and peptide-level *Q*-values (estimate using the target-decoy approach) across all repeats.
