# Supplementary figures and images for: Isolation and Characterization of Multi-Protein Complexes Enriched in the K-Cl Co-transporter 2 From Brain Plasma Membranes
Source: Front Mol Neurosci. 2020 Oct 22;13:563091. doi: 10.3389/fnmol.2020.563091 (PMC7643010; doi:10.3389/fnmol.2020.563091)

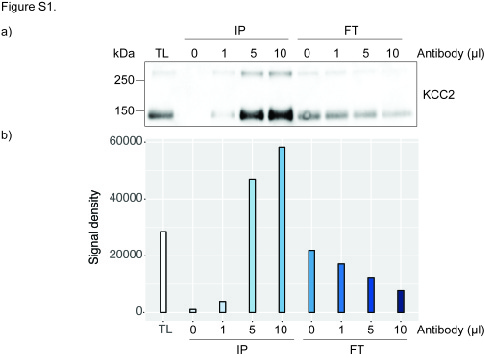

Supplement: FIGURE S1 — Initial optimization of antibody:bead ratios for optimal Kcc2 enrichment by immunoprecipitation. Immunoblots were carried out on protein resolved by SDS-PAGE. Total lysate (TL) levels of Kcc2 were compared with immunoprecipitated (IP) samples purified with 0–10 μl of Kcc2 antibody crosslinked to 50 μl of Protein G Dynabeads, and the respective flow-through (FT) samples (to detect the amount of Kcc2 extracted from the total pool). (B) Densitometry of the immunoblot demonstrating considerable enrichment of Kcc2 by immunoprecipitation, and depletion of approximately 65% of the total Kcc2 available at the highest antibody:bead ratio. [file Image_1.jpg]

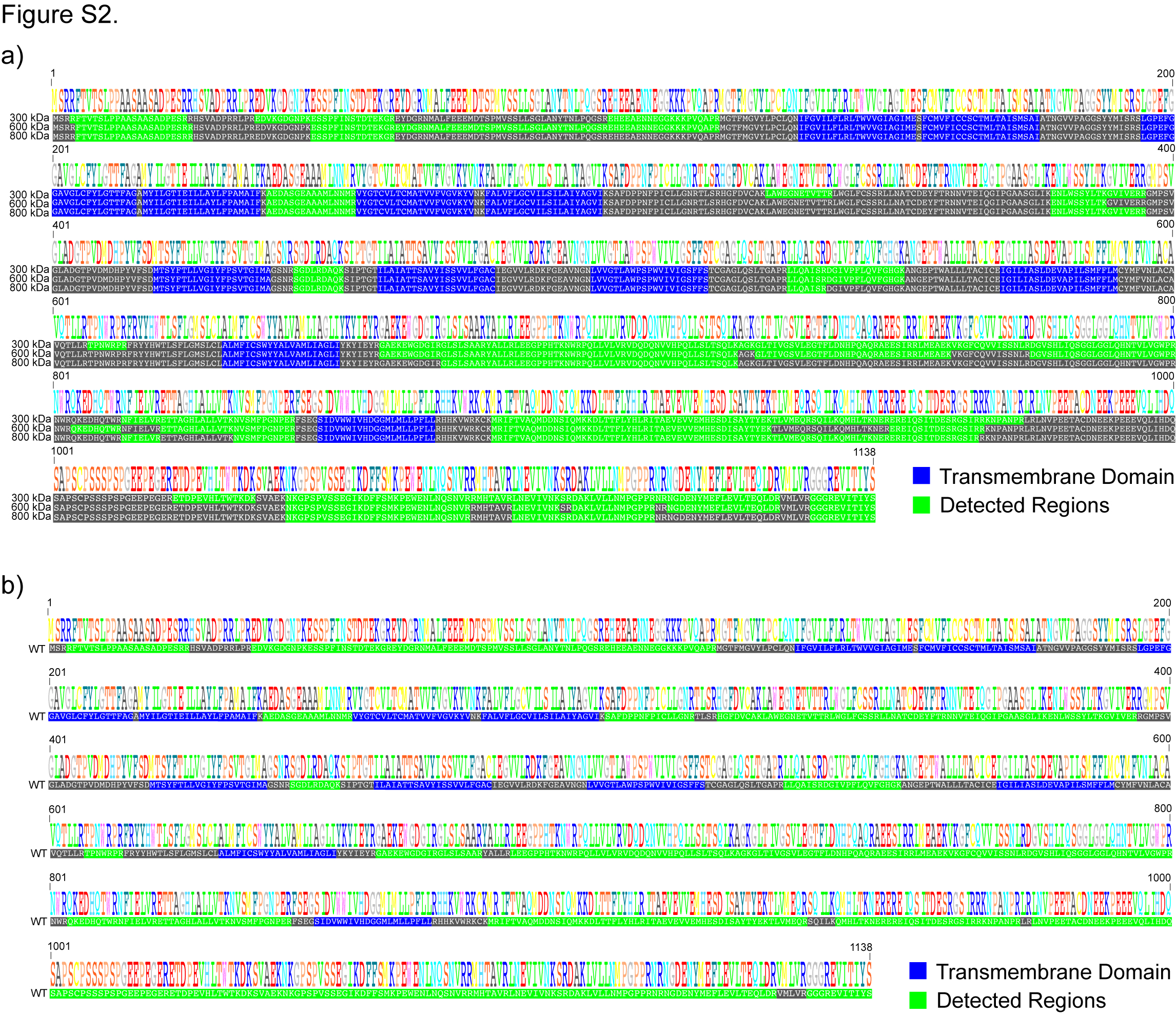

Supplement: FIGURE S2 — The total Kcc2 peptides detected by LC-MS/MS across all biological repeats were aligned to the Kcc2 reference sequence (UniProt ID: Q91V14) to visualize protein coverage. (A) The Kcc2 coverage obtained from the 300, 600, and 800 kDa bands of Kcc2 resolved by BN-PAGE. (B) The Kcc2 coverage of wild type (WT) mouse samples from the ∼140 kDa band resolved by SDS-PAGE. [file Image_2.jpg]
